# Anomaly Detection in High-Dimensional Time Series Data with Scaled Bregman Divergence

**DOI:** 10.3390/a18020062

**Published:** 2025-01-24

**Authors:** Yunge Wang, Lingling Zhang, Tong Si, Graham Bishop, Haijun Gong

**Affiliations:** 1 Department of Mathematics and Statistics, Saint Louis University, St. Louis, MO 63103, USA;; 2 Department of Mathematics and Statistics, University at Albany SUNY, Albany, NY 12222, USA;; 3 Mathematics Department, Culver-Stockton College, Canton, MO 63435, USA;

**Keywords:** anomaly detection, scaled Bregman divergence, density ratio estimation, least absolute deviation

## Abstract

The purpose of anomaly detection is to identify special data points or patterns that significantly deviate from the expected or typical behavior of the majority of the data, and it has a wide range of applications across various domains. Most existing statistical and machine learning-based anomaly detection algorithms face challenges when applied to high-dimensional data. For instance, the unconstrained least-squares importance fitting (uLSIF) method, a state-of-the-art anomaly detection approach, encounters the unboundedness problem under certain conditions. In this study, we propose a scaled Bregman divergence-based anomaly detection algorithm using both least absolute deviation and least-squares loss for parameter learning. This new algorithm effectively addresses the unboundedness problem, making it particularly suitable for high-dimensional data. The proposed technique was evaluated on both synthetic and real-world high-dimensional time series datasets, demonstrating its effectiveness in detecting anomalies. Its performance was also compared to other density ratio estimation-based anomaly detection methods.

## 1. Introduction

Anomaly detection (AD), also known as outlier detection, is the process of identifying data points, patterns, or events that deviate significantly from the expected or normal behavior within a dataset. Anomaly detection has a broad range of applications, including network security (e.g., detecting unauthorized access or cybersecurity threats) [1], fraud detection (e.g., unusual transactions in banking or insurance) [2], medical diagnostics (e.g., identifying tumors in MRI scans) [3], quality control in manufacturing (e.g., equipment faults), and healthcare and environmental monitoring (e.g., irregularities in vital signs of patients or unusual weather) [4]. Numerous anomaly detection methods based on statistical and machine learning algorithms have been developed and successfully applied across various fields [5,6]. These techniques aim to identify abnormal patterns within diverse types of datasets, including time series data.

Most existing anomaly detection techniques are generally categorized into supervised, semisupervised, and unsupervised approaches. The review by Salima et al. [7] provides a detailed discussion of various methods. Here, we briefly introduce some popular techniques for completeness. Supervised methods, also referred to as classification methods [8–10], rely on labeled training data to construct predictive models. Common supervised anomaly detection algorithms include Support Vector Machines (SVMs), k-Nearest Neighbors (k-NN), Bayesian Networks, Decision Trees, and Neural Networks. For example, Mafra et al. [11] proposed an intrusion detection system using Kohonen Neural Networks and SVM to identify anomalous patterns in network traffic. Similarly, SVMs have been combined with Decision Trees for intrusion detection [12] and Denial-of-Service (DoS) attack detection [13]. Neural Network-based approaches, such as Multi-Layer Perceptrons (MLPs) or Artificial Neural Networks, have also been employed to differentiate between normal and abnormal behaviors. For instance, Radial Basis Function Neural Networks (RBF) [14] were utilized to learn multiple local clusters for well-known attacks. These methods can also integrate results from multiple classifiers to enhance performance, making them particularly well suited for problems involving large datasets. Unsupervised anomaly detection methods identify anomalies based on the intrinsic characteristics of the data, without requiring any labeled training data or prior knowledge of which points are anomalous. These methods are based on the assumption that the majority of data points are normal, while only a small fraction are anomalous. Common unsupervised anomaly detection algorithms include clustering-based methods such as K-Means [15], Fuzzy C-Means [16], and the unsupervised niche clustering-based anomaly detection algorithm [17], as well as probabilistic approaches like the Expectation-Maximization (EM) Meta algorithm [18]. Deep learning-based techniques, such as Generative Adversarial Network (GAN)-based [19,20], variational autoencoder (VAE)-based [21–23], transformer-based [24–26], and diffusion model-based [27] anomaly detection approaches, have garnered significant attention due to their ability to model complex data distributions and identify subtle anomalies. Popular generative model-based tools for anomaly detection include MAD-GAN [20], VAE-GAN [23], UTRAD [26], etc. However, these methods often require a large amount of data for effective training, which can be a significant limitation in scenarios where data availability is restricted. This reliance on extensive training data poses challenges in applications with limited labeled or high-quality datasets. The semisupervised anomaly detection methods, for example, GANomaly [28], typically use a small set of labeled data, which provide information about normal or anomalous patterns, alongside a larger set of unlabeled data to learn the underlying data distribution and detect deviations.

Though significant progress has been made in anomaly detection algorithms, several challenges remain. Most existing anomaly detection methods require learning the underlying distribution of the data to differentiate between normal and anomalous data, which is challenging, particularly in cases where the data distribution is high-dimensional, non-stationary, or multimodal. Accurately capturing these distributions often demands complex models and large amounts of data, which may not always be available. Furthermore, the assumption of a specific distribution may not hold true for all datasets, potentially limiting the effectiveness of these methods. Although directly estimating individual distributions is challenging in high-dimensional data, estimating the ratio of the densities between training and test data [29,30] is comparatively more feasible. Density ratio estimation-based approaches circumvent the need for explicit distribution estimation by focusing on the relative likelihood of data points under different conditions. Techniques such as density ratio estimation [30,31] and ratio matching [32] methods offer an alternative for anomaly detection in complex, high-dimensional data since they simplify the problem by estimating the ratio between normal and anomalous data distributions.

Density ratio estimation involves directly estimating the ratio between the probability densities of two distributions, typically corresponding to normal (inlier) and anomalous (outlier) data. This approach avoids the explicit estimation of individual densities and focuses on identifying deviations between two distributions. Several techniques have been developed for density ratio estimation, including the Kullback–Leibler Importance Estimation Procedure (KLIEP) [29], unconstrained least-squares importance fitting (uLSIF) [31], Relative uLSIF (RuLSIF) [30], Bregman divergence-based [33], RobustRealNVP [34], and Pearson-like scaled Bregman divergence-based (PLsBD) [35] methods. In the previous studies [30,35,36], these approaches have been successfully applied to identify change points in time series data, a problem closely related to anomaly detection. Change-point detection, like anomaly detection, involves identifying significant shifts in data behavior over time. The existing density ratio estimation methods utilize various divergence measures such as the Kullback–Leibler (KL), Pearson, and α-relative Pearson divergence to quantify the dissimilarity between data distributions and detect the change points or anomalies. For example, Hido et al.’s work [37] applied uLSIF to identify anomalies; as we discussed above, these divergence functions may introduce severe unboundedness issues under some conditions. Moreover, most density ratio estimation methods adopt a squared error ($L_{2}$) loss as the optimization objective to estimate the density ratio [30,37–39]. This approach is widely favored for its simplicity and computational efficiency in convex optimization problems. However, its sensitivity to outliers and limitations in handling non-Gaussian noise can affect performance in certain applications.

To address the limitations of existing anomaly detection methods, we propose a scaled Bregman divergence-based anomaly detection approach with density ratios, inspired by our recent change-point detection work [35]. Compared to existing density ratio estimation-based methods, the proposed scaled Bregman divergence-based approach, using both $L_{1}$ and $L_{2}$ loss functions, addresses the unboundedness issue, ensuring stable divergence calculations. Additionally, compared to deep learning-based methods, our approach is more computationally efficient, particularly in handling high-dimensional datasets with limited data availability. The flexibility of scale-Bregman divergence can improve the accuracy and reliability of anomaly detection, particularly in high-dimensional or complex data. The rest of this paper is organized as follows: In the Materials and Methods section, we provide a detailed review of the scaled Bregman divergence-based density ratio anomaly detection approach, using both $L_{1}$ and $L_{2}$ loss functions as objective functions. In the Results section, we apply the proposed method to detect anomalies in both synthetic data and high-dimensional real-world datasets, comparing its performance with the uLSIF and RuLSIF methods. Finally, in the Discussion section, we examine the advantages and limitations of the proposed algorithm, as well as potential directions for future work.

## 2. Materials and Methods

An anomaly, or outlier, is an observation that significantly deviates from the majority of the data, often indicating that the distribution of data containing the anomaly differs from that of normal data. So, the anomaly detection aims to identify such patterns, which may signify critical information, by modeling the underlying probability distributions of the data and distinguishing anomalies from normal observations.

We use two datasets, a training set ${\left\{x_{j}^{tr}\right\}}_{j=1}^{n_{tr}}\sim p_{tr}(x)$, which consists entirely of normal samples, and a test set ${\left\{x_{i}^{\text{te}}\right\}}_{i=1}^{n_{te}}\sim p_{te}(x)$, which may include both normal and anomalous samples, where $p_{tr}(x)$ and $p_{te}(x)$ are the respective probability density functions. The training set is used to learn model parameters that capture the distribution of normal data, under the assumption that they do not contain any anomalies. By comparing the test data distribution against the learned normal data distribution, anomalies can be identified as data points that significantly deviate from the expected normal distribution. Individual density estimation is often impractical in high-dimensional data. In this work, we focus on the density ratio between the training and test data, $w(x)=\frac{p_{tr}(x)}{p_{te}(x)}$, which eliminates the need for the estimation of individual density functions. Next, we will discuss how to integrate the density ratio with divergence functions to develop a more effective framework for anomaly detection.

### 2.1. Scaled Bregman Divergence-Based Dissimilarity Measure

Previous studies have employed divergence measures such as Kullback–Leibler (KL) divergence and Pearson (PE) divergence to quantify the dissimilarity between two distributions, $p_{tr}(x)$ and $p_{te}(x)$. For instance, the Kullback–Leibler Importance Estimation Procedure (KLIEP) [29] was developed for anomaly detection and change-point detection using KL divergence. Similarly, Pearson divergence has been used in the unconstrained least-squares importance fitting (uLSIF) method [31], which has been applied to detect anomalies [37] and change points [40]. Pearson divergence is defined as

(1) $$PE\left(p_{tr}\|p_{te}\right)=\frac{1}{2}\int p_{te}\left(x\right){\left(\frac{p_{tr}\left(x\right)}{p_{te}\left(x\right)}-1\right)}^{2}dx.$$

Hido et al.’s work [37] demonstrated that uLSIF outperforms other algorithms for anomaly detection due to its unique global optimal solution. However, it has been observed in prior change-point detection studies that the density ratio used in uLSIF can become unbounded under certain conditions, potentially affecting the method’s stability.

The unboundedness problem commonly encountered when using KL and Pearson divergences as dissimilarity measures poses significant challenges in many anomaly detection and change-point detection applications [40]. To mitigate this issue, a RuLSIF (relative unconstrained least-squares importance fitting) method, which utilizes the α-PE divergence, was developed for change-point detection [40], but has never been applied for anomaly detection. Furthermore, our recent work [35] proposed a scaled Bregman divergence for change-point detection, which has shown superior performance in addressing these challenges. In this study, we extend and adapt this scaled Bregman divergence-based method for anomaly detection.

Given three measures P,Q and M on 𝒳 with densities p,q and m, respectively, a scaled Bregman divergence [41] is defined as

(2) $$D(p\left\|q;m\right)=\int_{𝒳}\left[f\left(\frac{p\left(x\right)}{m\left(x\right)}\right)-f\left(\frac{q\left(x\right)}{m\left(x\right)}\right)-f^{\prime }\left(\frac{q\left(x\right)}{m\left(x\right)}\right)\left(\frac{p\left(x\right)}{m\left(x\right)}-\frac{q\left(x\right)}{m\left(x\right)}\right)\right]m\left(x\right)dx,$$

where $f:R^{+}\to R$ is a convex function, and $f^{\prime }$ is its right derivative. Different choices of the measure M and function f yield various well-known divergence functions. For instance, let $f(t)=\frac{1}{n}(t-1{)}^{n}$; when n=2, a novel divergence, termed the Pearson-like scaled Bregman divergence (PLsBD), was derived in our recent work [35], which is expressed as

(3) $$PL\left(p_{tr}\|p_{te};m\right)=\frac{1}{2}\int_{𝒳}m(x){\left(\frac{p_{tr}(x)}{m(x)}-\frac{p_{te}(x)}{m(x)}\right)}^{2}dx.$$

Our work [35] has theoretically proven several properties of PLsBD compared to other divergences. For example, PLsBD is bounded, can be symmetric, and can serve as a metric measure under certain conditions. Consequently, in our change-point detection application, PLsBD has demonstrated superior performance compared to all density ratio estimation-based methods. It is easy to verify that if the measure density $m(x)=p_{te}(x)$, the proposed PLsBD reduces to the Pearson divergence.

To address the unboundedness issue in the divergence measure, we typically introduce a measure density $m_{\alpha }(x)=\alpha p_{tr}(x)+(1-\alpha )p_{te}(x)$, where the parameter α∈(0,1) controls the mixing of the training and testing distributions. This balancing of contributions from both distributions ensures stability in the divergence calculation and effectively mitigates unboundedness issues.

**Theorem 1.** *Given the measure* $m_{\alpha }(x)=\alpha p_{tr}(x)+(1-\alpha )p_{te}(x)$, *where* α∈(0,1), *the Pearson-like scaled Bregman divergence between* $p_{\text{tr}}$ and $p_{\text{te}}$ is defined as

$$PL\left(p_{tr}\|p_{te};m_{\alpha }(x)\right)=\frac{1}{2}\int m_{\alpha }\left(x\right){\left(\frac{p_{tr}\left(x\right)}{m_{\alpha }\left(x\right)}-\frac{p_{te}\left(x\right)}{m_{\alpha }\left(x\right)}\right)}^{2}dx,$$

*is bounded and can be expressed as* [35]:

$$PL\left(p_{tr}\|p_{te};m_{\alpha }(x)\right)=\frac{1}{2}E_{p_{tr}}\left[w_{\alpha }(x)\right]-\frac{2-\alpha }{2(1-\alpha )}E_{p_{te}}\left[w_{\alpha }(x)\right]+\frac{1}{2(1-\alpha )},$$

*where* $w_{\alpha }(x)=\frac{p_{tr}(x)}{m_{\alpha }(x)}$, *and* $E_{p}[\cdot ]$ *denotes the expectation with respect to the density* p(x).

The detailed proof of Theorem 1 can be found in our recent work [35]. The PLsBD divergence $PL\left(p_{tr}\|p_{te};m_{\alpha }(x)\right)$ can be approximately computed by the empirical average:

(4) $$\hat{PL}\left(p_{tr}\|p_{te};m_{\alpha }(x)\right)\approx \frac{1}{2n_{tr}}\sum_{i=1}^{n_{tr}}\overset{ˆ}{w}\left(x_{i}^{tr}\right)-\frac{2-\alpha }{2(1-\alpha )}\sum_{i=1}^{n_{te}}\overset{ˆ}{w}\left(x_{i}^{te}\right)+\frac{1}{2(1-\alpha )}.$$

It is worth noting that when n=3, it is straightforward to derive another, more complex divergence:

$$PL\left(p_{tr}\|p_{te};m\right)=\frac{1}{3}\int_{𝒳}m(x){\left(\frac{p_{tr}(x)}{m(x)}-\frac{p_{te}(x)}{m(x)}\right)}^{2}\left(\frac{p_{tr}(x)}{m(x)}+\frac{2p_{te}(x)}{m(x)}-3\right)dx.$$

However, this divergence is not utilized in our work, not only due to our preference for the simpler form obtained when n=2, but also because this divergence is not symmetric under all conditions.

The α-relative Pearson (α-PE) divergence is another divergence commonly used to quantify the discrepancy between two distributions. By replacing $p_{te}$ in the standard Pearson divergence with $p_{te,\alpha }(x)=\alpha p_{tr}(x)+(1-\alpha )p_{te}(x)$, which represents a mixture of the training and test densities, we obtain the α-PE divergence, which can be viewed as a specific instance of the scaled Bregman divergence. This modification addresses the unboundedness problem associated with the standard Pearson divergence. It is defined as

$$PE_{\alpha }\left(p_{tr}\|p_{te,\alpha }\right)=\frac{1}{2}\int p_{te,\alpha }\left(x\right){\left(\frac{p_{tr}\left(x\right)}{p_{te,\alpha }\left(x\right)}-1\right)}^{2}dx,\;=\frac{1}{2}E_{p_{tr}}\left(w_{\alpha }(x)\right)-\frac{1}{2}.$$

The α-PE divergence $PE_{\alpha }\left(p_{tr}\|p_{te,\alpha }\right)$ can be approximately computed by the empirical average:

(5) $$\hat{PE}\left(p_{tr}\|p_{te};p_{te,\alpha }(x)\right)\approx \frac{1}{2n_{tr}}\sum_{i=1}^{n_{tr}}\overset{ˆ}{\omega }\left(x_{i}^{tr}\right)-\frac{1}{2}.$$

RuLSIF (relative unconstrained least-squares importance fitting) applied the α-PE divergence in previous studies for change-point detection to overcome the unboundedness issue, but has not been applied for the anomaly detection. However, α-PE measures the dissimilarity between $p_{tr}$ and the mixture distribution $p_{te,\alpha }$, rather than between two distinct distributions. Our work [35] has shown that the RuLSIF method is highly sensitive to the choice of α, with large α values potentially introducing numerical instability. The PLsBD directly measures the disparity between two individual distributions, rather than a mixture, making it more robust and reliable in applications such as change-point detection. As shown by the empirical averages in Equations (4) and (5), PLsBD demonstrates greater effectiveness in anomaly detection by incorporating data from both training and test distributions for divergence estimation. This approach enhances its ability to detect subtle anomalies arising from shifts in the test data, albeit at the cost of increased computational complexity. In contrast, the α-PE divergence relies predominantly on the training data, reducing its sensitivity to anomalies that emerge exclusively in the test distribution. In this work, we will apply both PLsBD and RuLSIF methods for anomaly detection and compare their performance with the uLSIF-based anomaly detection method.

### 2.2. Parameter Learning by Least Squares and Least Absolute Deviation

The density ratio $w_{\alpha }(x)=\frac{p_{tr}(x)}{p_{te,\alpha }(x)}$ can be estimated by an estimator $\overset{ˆ}{w}(x;\theta )$, which is modeled as a Gaussian kernel regression [36]:

(6) $$\overset{ˆ}{w}\left(x;\theta \right)=\sum_{l=1}^{n}\theta_{l}K_{\sigma }\left(x,x_{l}\right),$$

where $\theta ={\left(\theta_{1},\theta_{2},\ldots ,\theta_{n}\right)}^{T}$ are parameters learned from the data, and $K_{\sigma }\left(x,x^{\prime }\right)$ is a Gaussian kernel expressed as $K_{\sigma }\left(x,x^{\prime }\right)=\text{exp}\;\left(-\frac{{\left\|x-x^{\prime }\right\|}^{2}}{2\sigma^{2}}\right)$ with kernel width σ(>0). In this paper, the kernel width σ is determined using a cross-validation method. Next, we will discuss how to learn the parameters θ using least absolute deviation and least squared loss methods.

#### 2.2.1. Least Absolute Deviation

The optimal θ can be learned by minimizing either the absolute error/deviation or the squared loss between the true and estimated relative ratios. The absolute error is defined as

(7) $$J_{L_{1}}\left(x\right)=\int \left|w_{\alpha }\left(x\right)-\overset{ˆ}{w}\left(x;\theta \right)\right|p_{te,\alpha }\left(x\right)dx,$$

which can be expressed as

(8) $$J_{L_{1}}\left(x\right)=\left|1-\alpha \int \overset{ˆ}{w}\left(x\right)p_{tr}\left(x\right)dx-\left(1-\alpha \right)\int \overset{ˆ}{w}\left(x\right)p_{te}\left(x\right)dx\right|.$$

Minimizing the absolute error loss is equivalent to solving the following convex optimization problem:

(9) $$\overset{ˆ}{\theta }=\underset{\theta \in R^{n}}{\text{argmin}}\left|1-\alpha {\hat{h}}_{tr}^{⊤}\theta -(1-\alpha ){\hat{h}}_{te}^{⊤}\theta \right|,$$

where ${\hat{h}}_{tr}$ and ${\hat{h}}_{te}$ are n-dimensional vectors with the l-th element given by

(10) $${\left({\hat{h}}_{tr}\right)}_{l}=\frac{1}{n_{tr}}\sum_{i=1}^{n_{tr}}K_{il}^{tr};\;{\left({\hat{h}}_{te}\right)}_{l}=\frac{1}{n_{te}}\sum_{j=1}^{n_{te}}K_{jl}^{te};$$

and $K_{il}^{tr/te}=K\left(Y_{i}^{tr/te},Y_{\ell };\sigma \right)$ represents the Gaussian kernel evaluated for the training or test samples.

The global optimal solution $\overset{ˆ}{\theta }$ for the least absolute deviation problem can be obtained analytically as

(11) $$\overset{ˆ}{\theta }={\left[\alpha {\hat{h}}_{tr}+(1-\alpha ){\hat{h}}_{te}\right]}^{-1}.$$

#### 2.2.2. Least Squared Loss

The least squared loss method has been discussed in previous works [35,40], and an analytical solution is available. For comparison, we will briefly review the results. The squared loss is defined as

(12) $$J_{L_{2}}(x)=\frac{1}{2}\int {\left(w_{\alpha }(x)-\overset{ˆ}{w}(x;\theta )\right)}^{2}p_{te,\alpha }(x)dx.$$

Including an $\ell_{2}$-regularization term, minimizing the squared loss is equivalent to solving the following convex optimization problem:

(13) $$\overset{ˆ}{\theta }=\underset{\theta \in R^{n}}{\text{argmin}}\left[\frac{1}{2}\theta^{⊤}\hat{H}\theta -{\hat{h}}_{tr}^{⊤}\theta +\frac{\lambda }{2}\theta^{⊤}\theta \right],$$

where $\hat{H}$ is an n×n matrix with the $\left(\ell ,\ell^{\prime }\right)$-th element, same as the results in [35,40]:

(14) $$(\hat{H}{)}_{\ell ,\ell^{\prime }}=\frac{\alpha }{n_{tr}}\sum_{i=1}^{n_{tr}}K_{il}^{tr}K_{il^{\prime }}^{tr}+\frac{\left(1-\alpha \right)}{n_{te}}\sum_{j=1}^{n_{te}}K_{jl}^{te}K_{jl^{\prime }}^{te}.$$

The global optimal solution θ for least squared loss can be analytically derived as

(15) $$\overset{ˆ}{\theta }={\left(\hat{H}+\lambda I_{n}\right)}^{-1}h_{tr\prime }$$

where $I_{n}$ denotes an n-dimensional identity matrix.

### 2.3. Algorithm and Computational Complexity Analysis

#### 2.3.1. Anomaly Detection Algorithm

Algorithm 1 outlines the procedure for anomaly detection using the proposed PLsBD and α-relative Pearson divergence (α-PE), incorporating both $L_{1}$ and $L_{2}$ loss functions for parameter learning. This algorithm is adapted from the change-point detection method described in our recent work [35], with a key difference: in anomaly detection, the training data window remains fixed, whereas in change-point detection, both the training and testing data windows slide simultaneously from left to right.

The anomaly detection process involves four main steps:

1. **Data Preparation:** The dataset is divided into training and test sets. The training set consists only of normal samples, while the test set may include anomalies. The algorithm computes distribution divergences between the training and test samples by sliding a window from left to right.
2. **Parameter Optimization:** Cross-validation is used to determine the optimal values of parameters, including kernel width (σ) and regularization term (λ).
3. **Density Ratio Estimation:** Both the least absolute deviation $\left(L_{1}\right)$ and least-squares loss ($L_{2}$) approaches employ kernel computations to compute the optimal coefficients (θ), which are then used to estimate density ratios.
4. **Anomaly Identification:** Divergence scores, either PLsBD or α-PE, are calculated, and samples are flagged as potential anomalies if their scores exceed a predefined percentile threshold of the maximum score. Then, the sliding window is moved to the right, and the procedure is repeated.

Notably, setting α=0 reduces the method to the unconstrained least-squares importance fitting (uLSIF), a state-of-the-art technique for density ratio estimation and anomaly detection [37].

**Algorithm 1:** Anomaly detection using PLsBD and αPE measures with both $L_{1}$ and $L_{2}$ loss functions

**Input:** Training samples ${\left\{x_{j}^{tr}\right\}}_{j=1}^{n_{tr}}$, test samples ${\left\{x_{i}^{te}\right\}}_{i=1}^{n_{te}}$, number of folds in cross-validation k, mixing parameter α, number of basis functions $n_{b}$, threshold τ∈[0,1].
**Output:** Anomaly candidates.
**Step 1: Optimal Parameter Selection via Cross-Validation** For each σ,λ value, For each σ and λ, perform k-fold cross-validation: – Compute the kernel matrix $K_{\sigma }\left(x,x^{\prime }\right)$. – Calculate ${\hat{h}}_{tr},{\hat{h}}_{te}$, and $\hat{H}$. Apply $n_{f}$-fold cross validation to identify optimal σ and λ.
**Step 2: Estimator Learning by Solving Optimization Problems** Using the optimal values of σ and λ, For the $L_{1}$ loss-based optimization, solve $$\hat{\theta }={\left(\alpha {\hat{h}}_{tr}+(1-\alpha ){\hat{h}}_{te}\right)}^{-1},$$ For the $L_{2}$ loss-based optimization problem, solve $$\hat{\theta }={\left(\hat{H}+\lambda I_{n}\right)}^{-1}{\hat{h}}_{tr},$$
**Step 3: Calculation of Divergence Score** Compute the estimated density ratio $\hat{w}(x)=\sum_{i=1}^{n_{tr}}{\hat{\theta }}_{i}K_{\sigma }\left(x,x_{i}^{tr}\right)$. Calculate the divergence between training and testing data: – PLsBD divergence: $$\hat{PL}\left(p_{tr}\|p_{te};m_{\alpha }\right)\approx \frac{1}{2n_{tr}}\sum_{i=1}^{n_{tr}}\hat{w}\left(x_{i}^{tr}\right)-\frac{2-\alpha }{2(1-\alpha )}\sum_{i=1}^{n_{te}}\hat{w}\left(x_{i}^{te}\right)+\frac{1}{2(1-\alpha )}.$$ – α-relative PE divergence: $$\hat{\alpha PE}\left(p_{tr}\|p_{te};p_{te,\alpha }(x)\right)\approx \frac{1}{2n_{tr}}\sum_{i=1}^{n_{tr}}\hat{w}\left(x_{i}^{tr}\right)-\frac{1}{2}.$$
**Step 4: Identify Anomaly Candidates** Identify the maximum divergence score: ${\hat{PL}}_{\text{max}}$ or ${\hat{\alpha PE}}_{\text{max}}$. An anomaly is detected if: $$\frac{\hat{PL}\left(p_{tr}\|p_{te};m_{\alpha }(x)\right)}{PL_{\text{max}}}>\tau \;\text{or}\;\frac{\hat{\alpha PE}\left(p_{tr}\|p_{te};p_{te,\alpha }(x)\right)}{\alpha PE_{\text{max}}}>\tau .$$

#### 2.3.2. Complexity Analysis of Divergence Calculation

In the comparison between Equations (4) and (5), the computation of the empirical average for the PLsBD requires $𝒪\left(n_{tr}+n_{te}\right)$ operations, while the empirical estimation of the α-PE divergence requires only $𝒪\left(n_{tr}\right)$ operations. Although PLsBD incurs a slightly higher cost during empirical averaging, this overhead is negligible compared to the cost of density ratio estimation, which will be analyzed below.

#### 2.3.3. Complexity Analysis of Density Ratio Estimation

Our method is based on a kernel-based density ratio estimation framework, which has computational complexity comparable to the uLSIF method analyzed by Kanamori et al. [42]. The optimization problems can be solved using either $L_{1}$ or $L_{2}$ loss functions, which differ in computational complexities.

##### Computational Complexity of $L_{2}$ Loss:

The computational complexity of the $L_{2}$ loss can be broken down into the following components:

- Kernel Matrix Computation: For $n_{tr}$ training samples, $n_{te}$ test samples, and an n×n kernel matrix, each kernel evaluation takes 𝒪(d), where d is the feature dimensionality in the data. The total complexity is
$$𝒪\left(n\cdot \left(n_{tr}+n_{te}\right)\cdot d\right).$$
- Matrix Inversion: Solving ${\left(\hat{H}+\lambda I_{n}\right)}^{-1}$ has a complexity of $𝒪\left(n^{3}\right)$.
- Vector Multiplication: Multiplying the inverted matrix with the n-dimensional vector ${\hat{h}}_{tr}$ requires $𝒪\left(n^{2}\right)$.

Thus, the total computational complexity for $L_{2}$ loss is dominated by the kernel matrix computation and matrix inversion:


$$𝒪\left(n\cdot \left(n_{tr}+n_{te}\right)\cdot d+n^{3}\right).$$

##### Computational Complexity of $L_{1}$ Loss-based Method:

The $L_{1}$ loss avoids matrix inversion, requiring only element-wise computations and vector additions. Its complexity is therefore determined solely by the kernel matrix computation:

$$𝒪\left(n\cdot \left(n_{tr}+n_{te}\right)\cdot d\right).$$

In summary, the additional $𝒪\left(n^{3}\right)$ term in $L_{2}$ loss, arising from matrix inversion, renders it significantly more computationally expensive for large n, particularly in high-dimensional kernel setups. In contrast, the $L_{1}$ loss provides a more efficient alternative while retaining comparable performance in many scenarios. Our subsequent real-world data analysis confirmed this observation: the $L_{2}$-loss-based method requires considerably more computation time than the $L_{1}$-loss-based method, while the influence of divergences on overall performance is negligible.

## 3. Results

In this section, we will implement Algorithm 1 to evaluate the performance of the proposed and existing anomaly detection algorithms on both synthetic and real-world datasets. Specifically, we compare the performance of uLSIF, RuLSIF, and PLsBD, using both the least absolute deviation and least squared loss approaches.

### 3.1. Parameter Configuration

In all experiments, the number of basis functions, represented by Gaussian kernels, is fixed at 50. Our preliminary studies indicated that 50 kernel functions are sufficient to effectively fit the model while maintaining a balance between computational cost and performance. The centers of the basis functions are randomly initialized from the test dataset, and 5-fold cross-validation method is employed to determine the optimal values of σ and λ. The value of α is typically chosen in the range of 0.01 to 0.1. In our studies, we select α=0.1 in most cases because this choice achieves a good balance between the training and testing distributions, ensuring stable and robust divergence calculations. Larger α values tend to dilute the algorithm’s ability to detect anomalies, as observed in the subsequent parameter sensitivity studies and consistent with Liu et al.’s findings [40].

A sliding window approach is employed, with the window size for testing data fixed at 3 and the step size at 1, based on our preliminary studies for consistency analysis. These parameters may be adjusted to suit specific datasets and application requirements. The threshold τ is set to a value of 0.9, representing the percentile above which a score is classified as a potential anomaly. The choice of τ depends on the specific application and the acceptable trade-off between false positives and false negatives. A larger τ value results in fewer identified anomalies, focusing on high-confidence detections; therefore, it is often preferred in high-stakes scenarios to minimize false alarms. Conversely, a smaller τ value may classify a larger number of points as anomalies, potentially increasing false positives. This makes smaller τ values more suitable for exploratory or less critical analyses, where capturing more potential anomalies for further investigation is a priority.

### 3.2. Simulation Dataset

#### Dataset 1: 1-dimensional data.

We generate 300 observations, in which 298 data points were sampled from standard normal distribution, 𝒩(0,1), and 2 anomalies were introduced at indices 34 and 68, which were generated from a shifted normal distribution, 𝒩(10,1), with values 15 and 20, respectively. The first $n_{tr}=200$ observations were taken as training data.

**Figure 1.**
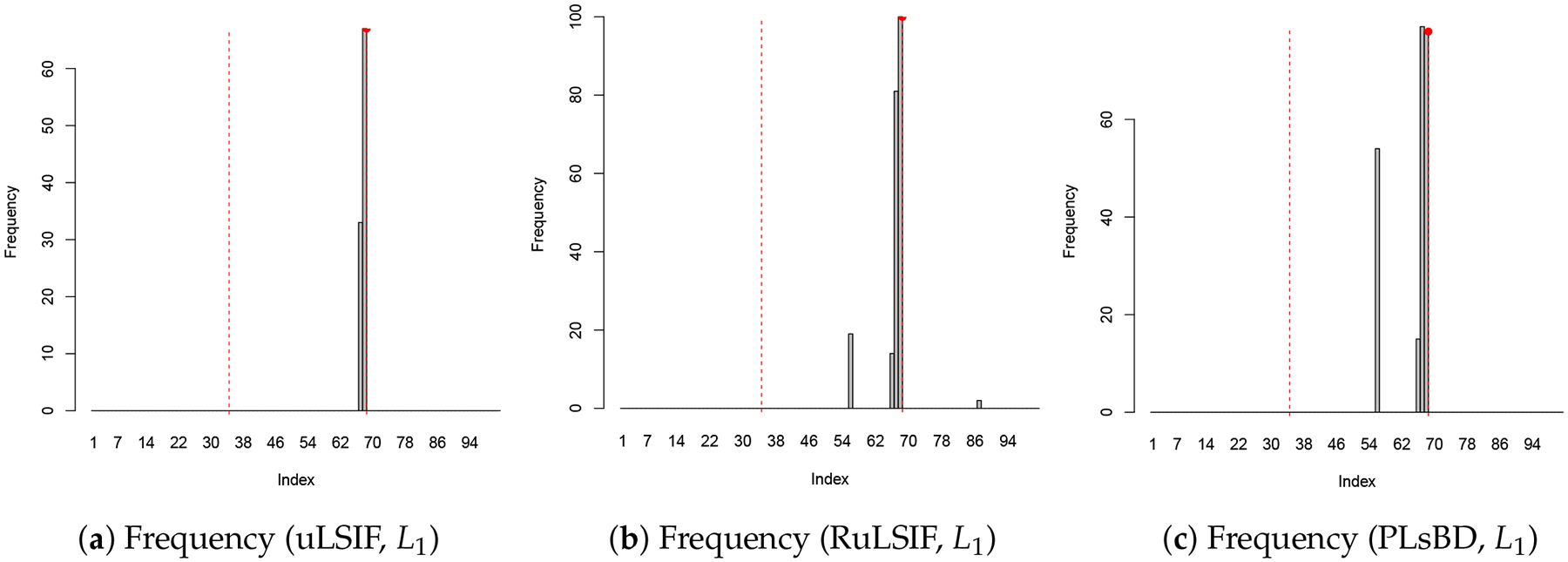

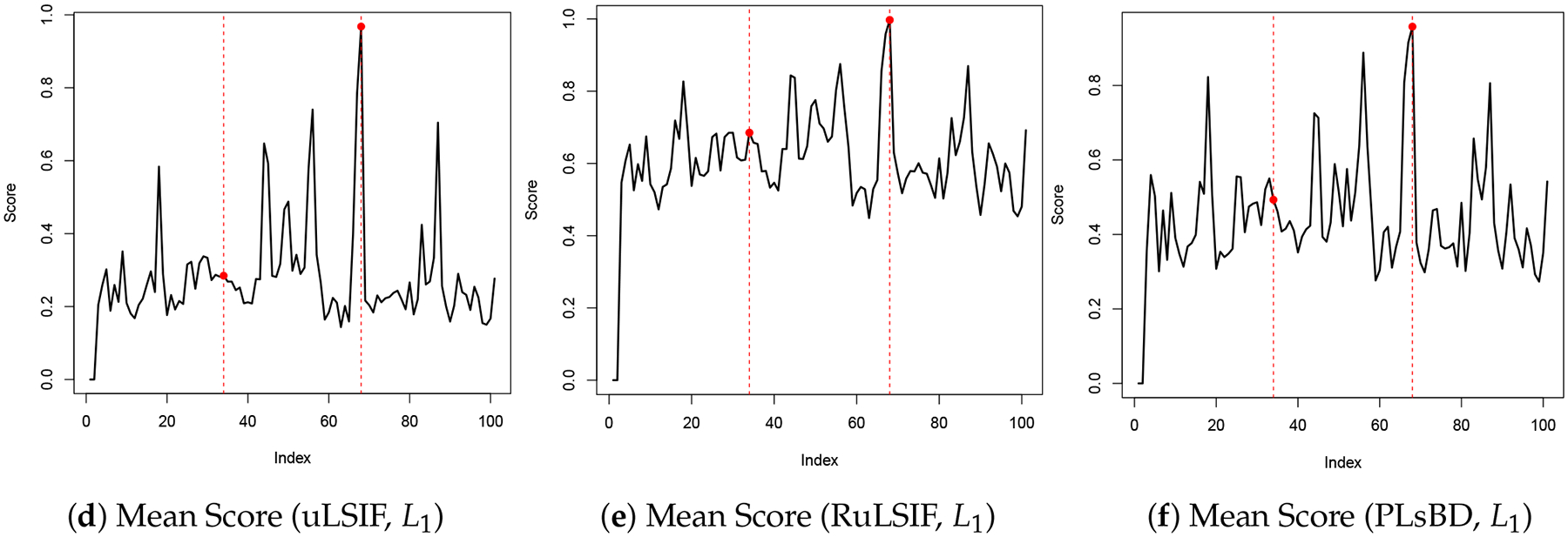
Anomaly detection results in Dataset 1 using uLSIF, RuLSIF, and PLsBD methods with $L_{1}$ loss function, and the mixing parameter α is set to be 0.1 for RuLSIF and PLsBD. The black bars indicate the frequencies that an observation is flagged as an anomaly in 100 runs. Red dashed vertical lines mark the true anomaly indices (34, 68).

**Figure 2.**
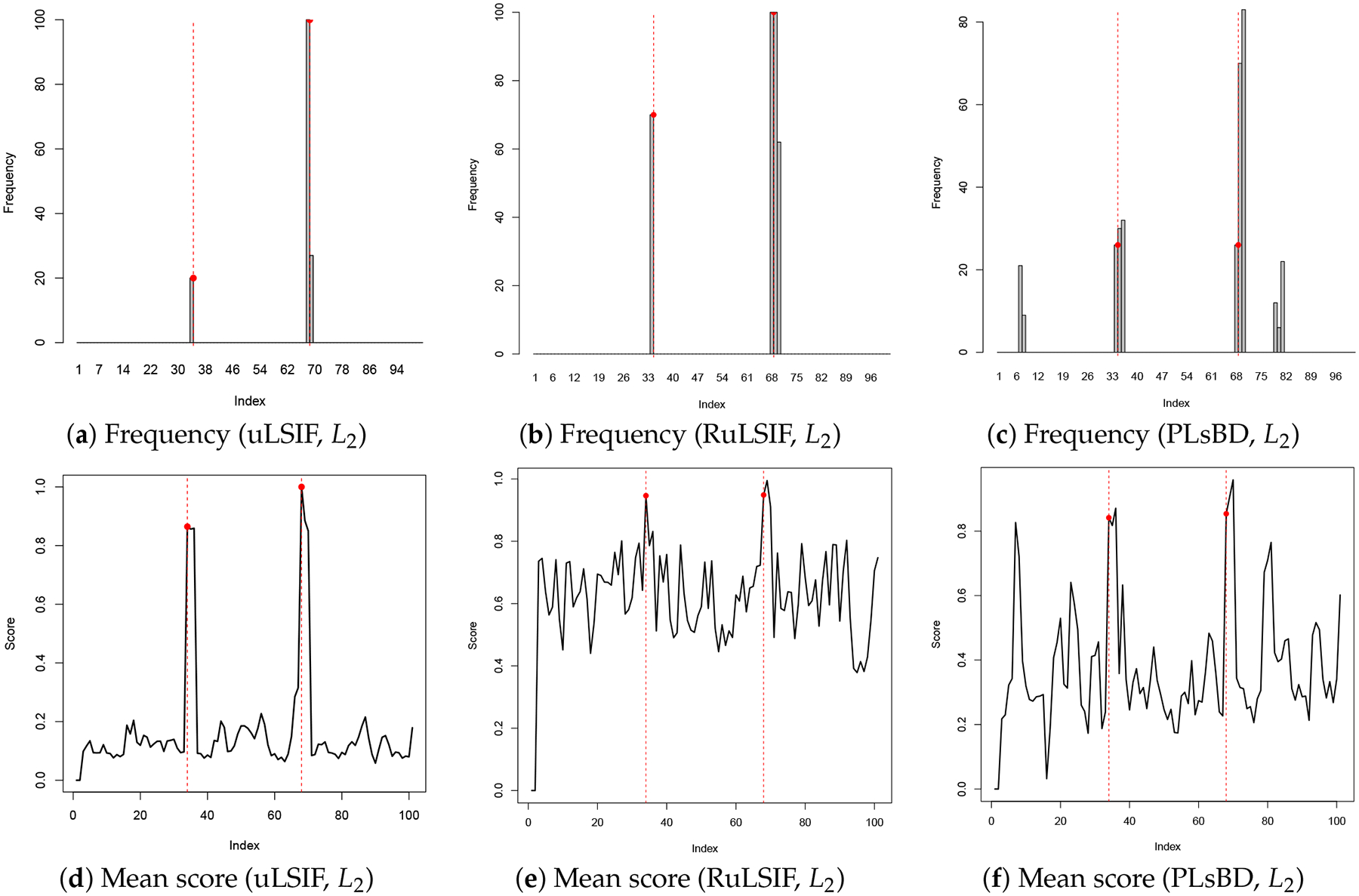
Anomaly detection results in Dataset 1 using uLSIF, RuLSIF, and PLsBD methods with the $L_{2}$ loss function, and the mixing parameter α is set to be 0.1 for RuLSIF and PLsBD. The black bars indicate the frequencies that an observation is flagged as an anomaly in 100 runs. Red dashed vertical lines mark the true anomaly indices (34, 68).

Figures 1 and 2 illustrate the frequency of anomaly candidates and their mean dissimilarity scores across 100 repeated runs. The results are presented for the uLSIF (a, d), RuLSIF (b, e), and PLsBD (c, f) measures. Both the $L_{1}$ (Figure 1) and $L_{2}$ (Figure 2) loss function methods are evaluated. The mixing parameter α is set to 0 for uLSIF, and 0.1 for RuLSIF and PLsBD. In the frequency plots, black bars indicate how often an observation in the test dataset is flagged as an anomaly in 100 runs. Red dashed vertical lines mark the true anomaly indices (34, 68). In the mean dissimilarity score plots, the black curve shows the mean normalized anomaly scores for each candidate, with red dashed vertical lines marking the true anomaly indices.

Figure 1 demonstrates that when using the absolute error as the loss function, only the second anomaly is detected by the uLSIF, RuLSIF, and PLsBD methods. However, as shown in Figure 2, employing the squared error as the loss function enables uLSIF, RuLSIF, PLsBD to identify both anomalies. This highlights the superior performance of the scaled Bregman divergence-based anomaly detection methods (PLsBD and RuLSIF), which successfully detect the outliers at indices 34 and 68. The observations in Figures 1 and 2 suggest that the $L_{2}$ loss is more effective in detecting anomalies than the $L_{1}$ loss. $L_{1}$ loss can detect more pronounced anomalies like the one at index 68, which appears less sensitive to subtle outliers such as the one at index 34. The squared error tends to yield larger values when anomalies are present compared to the absolute error, which enhances its sensitivity to outliers. This explains why the least squared loss has been widely adopted in anomaly detection and change-point detection in prior studies.

**Figure 3.**
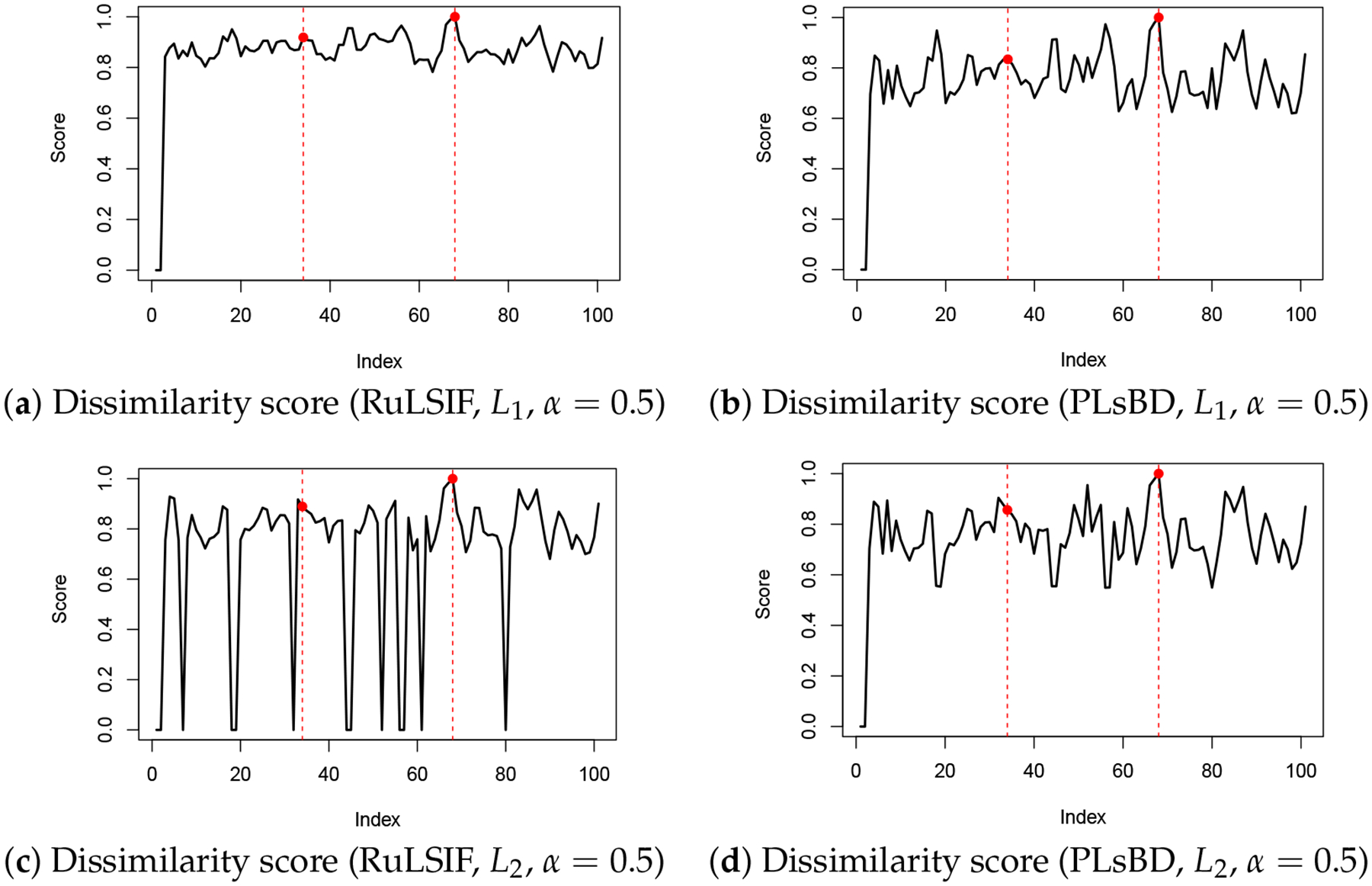
Dissimilarity score plots using RuLSIF and PLsBD methods with $L_{1}$ and $L_{2}$ loss functions, employing a mixing parameter of α=0.5. The analysis was conducted in a single run on Dataset 1. Red dashed vertical lines mark the true anomaly indices.

Our findings demonstrate that the performance of RuLSIF and PLsBD is sensitive to the choice of the mixing parameter α. Figure 3 presents the dissimilarity scores computed using RuLSIF and PLsBD with α=0.5. Notably, Figure 3c reveals significant fluctuations in the dissimilarity score when employing the $L_{2}$ loss function with RuLSIF, whereas PLsBD exhibits comparatively greater stability under the same conditions. The introduction of the mixing distribution aims to address the unboundedness issue; however, a larger mixing rate indicates that the testing data contain too much influence from the training data, potentially diluting the ability to detect anomalies or distributional differences effectively, eventually increasing false positives. Therefore, we selected a smaller α value for our subsequent data analysis to enhance performance.

#### Dataset 2: 2-dimensional data.

Similar to Dataset 1, we generate 300 samples of 2-dimensional data drawn from a multivariate normal distribution 𝒩(0,I), where **0** is the zero mean vector, and **I** is the identity covariance matrix. Two anomalies are introduced at indices 40 and 80 in the test set, with anomaly values shifted to **10** in both dimensions.

**Figure 4.**
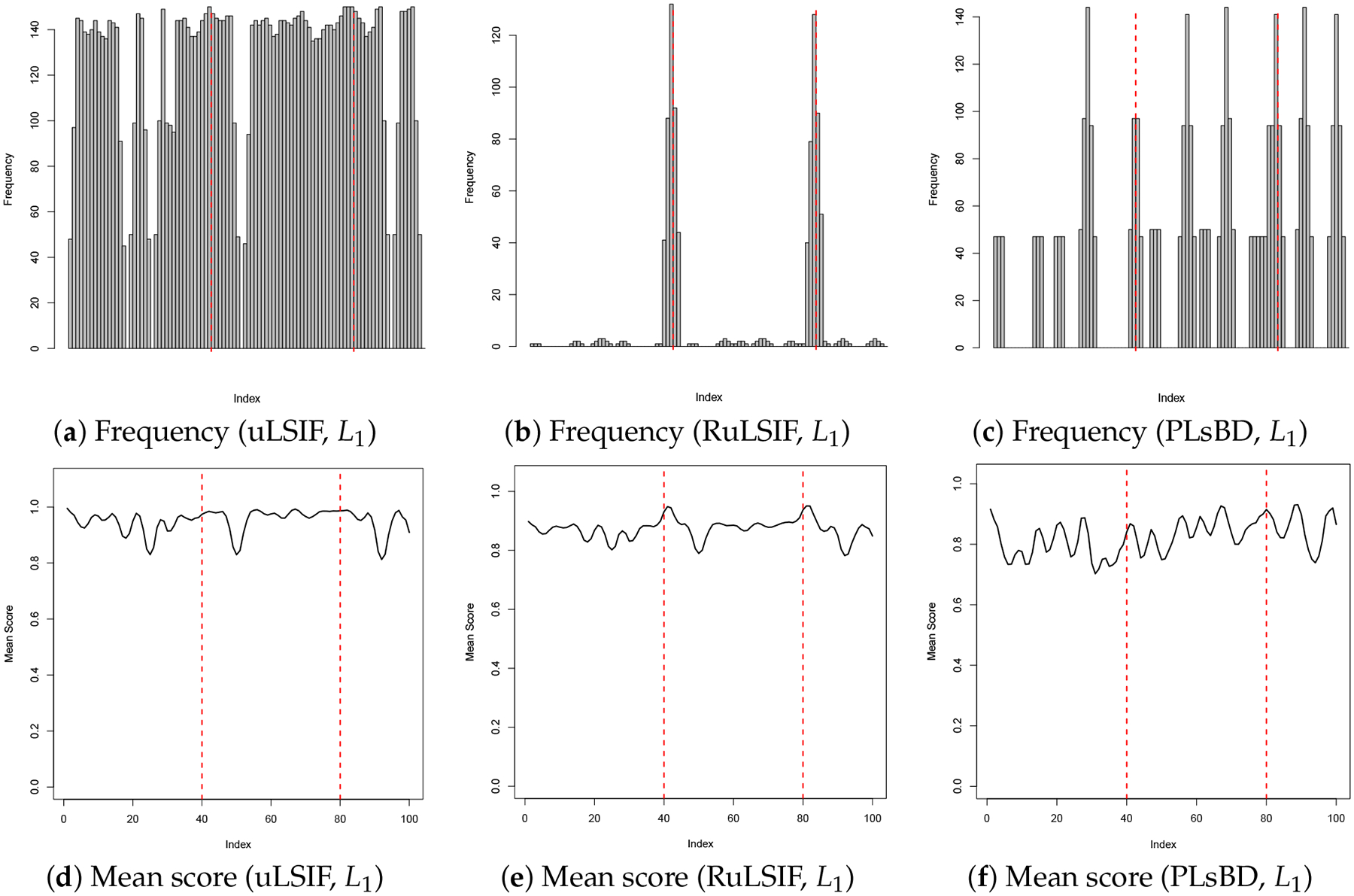
Anomaly detection results in Dataset 2 using uLSIF, RuLSIF, and PLsBD methods with the $L_{1}$ loss function, and the mixing parameter α is set to be 0.01 for RuLSIF and PLsBD. The black bars indicate the frequencies that an observation is flagged as an anomaly in 100 runs. Red dashed vertical lines mark the true anomaly indices (40, 80).

Figure 4 illustrates the frequency of anomaly candidates and their mean dissimilarity scores across 100 repeated runs using the uLSIF (a, d), RuLSIF (b, e), and PLsBD (c, f) measures, with parameters learned via the $L_{1}$ loss function. Figure 4a,d reveals that the uLSIF method exhibits the weakest performance in anomaly detection. Numerous data points are incorrectly identified as anomalies in the frequency plot, while the mean dissimilarity score remains flat and fails to provide meaningful insights. In contrast, the introduction of a small mixing parameter α=0.01 in RuLSIF significantly enhances its performance. While PLsBD also identifies the two true anomalies, RuLSIF outperforms PLsBD by producing fewer false positives. This is evident in Figure 4b,e, where RuLSIF displays two distinct peaks in the mean score plot, accurately corresponding to the true anomalies at indices 40 and 80.

**Figure 5.**
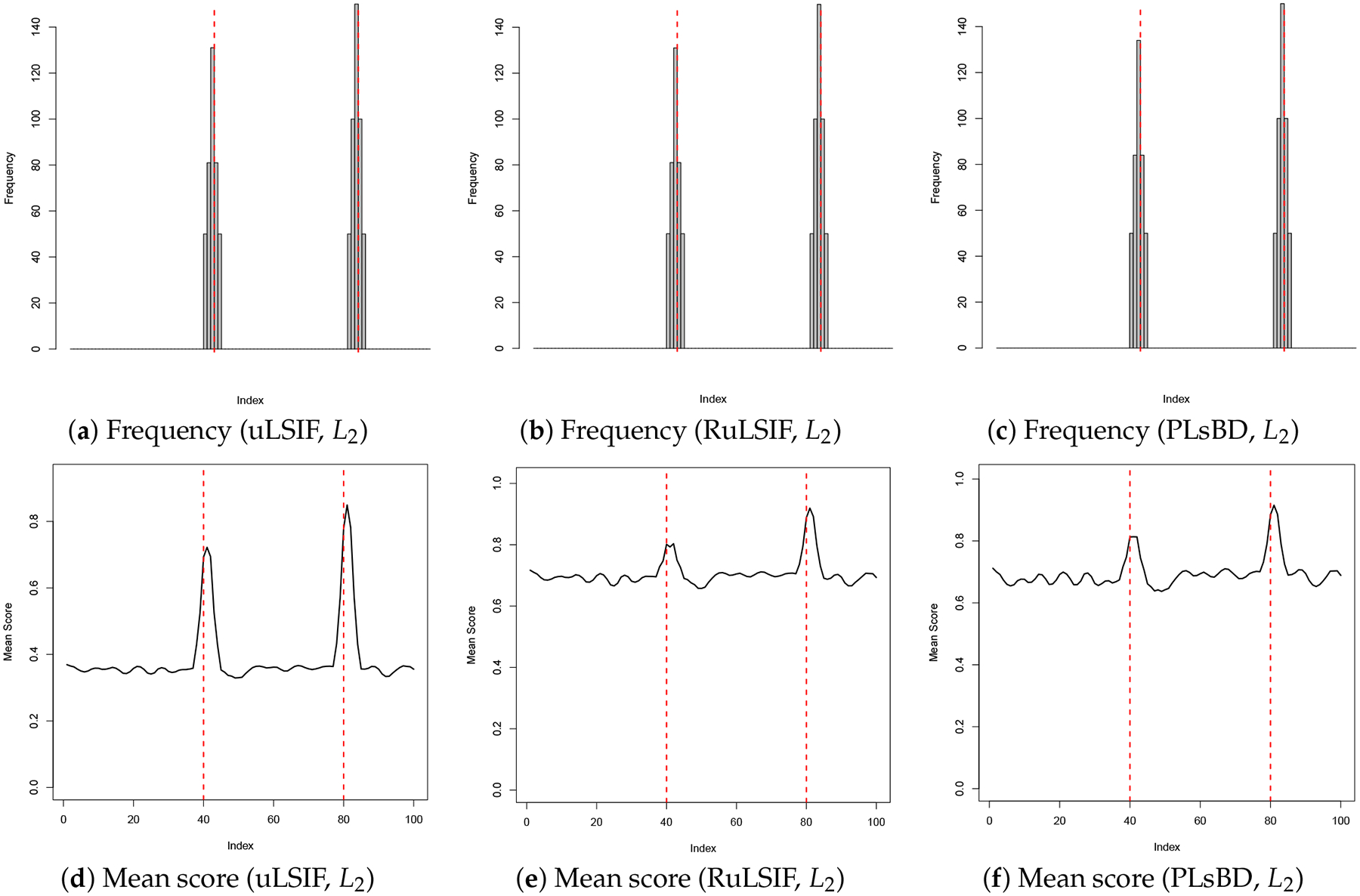
Anomaly detection results in Dataset 2 using uLSIF, RuLSIF, and PLsBD methods with the $L_{2}$ loss function, and the mixing parameter α is set to be 0.01 for RuLSIF and PLsBD. The black bars indicate the frequencies that an observation is flagged as an anomaly in 100 runs. Red dashed vertical lines mark the true anomaly indices (40, 80).

When using squared error as the loss function, as shown in Figure 5, all methods, uLSIF, RuLSIF, and PLsBD, demonstrate similar performance, consistently identifying the injected anomalies at indices 40 and 80 with minimal false positives. This result further supports the conclusion that the $L_{2}$ loss is more effective for anomaly detection compared to the $L_{1}$ loss. It is important to note that, since we employ a sliding window approach, an outlier candidate is treated as successfully detected if it falls within the interval [i-windowsize+1,i]. Our studies also indicate that larger window sizes and step sizes can decrease the sensitivity and overall performance of all anomaly detection algorithms. Consequently, in our studies, we have opted to use smaller window and step sizes to optimize detection sensitivity.

In summary, the findings from Figures 1–5 indicate that anomaly detection using the $L_{2}$ loss is generally more reliable than the $L_{1}$ loss in most cases with both RuLSIF and PLsBD methods. Our subsequent data analysis continues to reveal that when the dataset contains fewer anomalies, the $L_{2}$ loss function outperforms the $L_{1}$ loss. However, the performance gap between the two loss functions diminishes significantly when the data contain many anomalies. Furthermore, RuLSIF and PLsBD consistently outperform uLSIF, which is a state-of-the-art anomaly detection method, in both scenarios, highlighting their superior performance in anomaly detection tasks.

Our next step is to apply Algorithm 1 to analyze real-world low- and high-dimensional data, and to evaluate their performance in more complex, practical settings.

### 3.3. Real-World Datasets

#### Yahoo S5 A2Benchmark Dataset.

The Yahoo S5 benchmark dataset is widely used for evaluating anomaly detection methods, focusing on trend analysis and identifying deviations from normal patterns. It includes multiple one-dimensional time series datasets, and each dataset has three columns: timestamp (in Unix format), value (numerical measurements), and is.anomaly (binary label: 0 for normal, 1 for anomaly). In this experiment, we focus on the synthetic data 11 from the A2Benchmark datasets, a subset of the Yahoo S5 benchmark. The values in synthetic data 11 exhibit a periodic cyclical trend with regular fluctuations.

In our analysis, we use a subset of values from indices 450 to 550 as the training set, as it contains only normal data. The test set includes time points from indices 601 to 701, with anomalies occurring at indices 624–626 and 659–661. Given their close proximity, we treat indices 624–626 and 659–661 as two distinct anomaly intervals. The size of the sliding window is set to 3 and step size to 1, which are the same as the simulation data analysis, and all the experiments are repeated 50 times.

**Figure 6.**
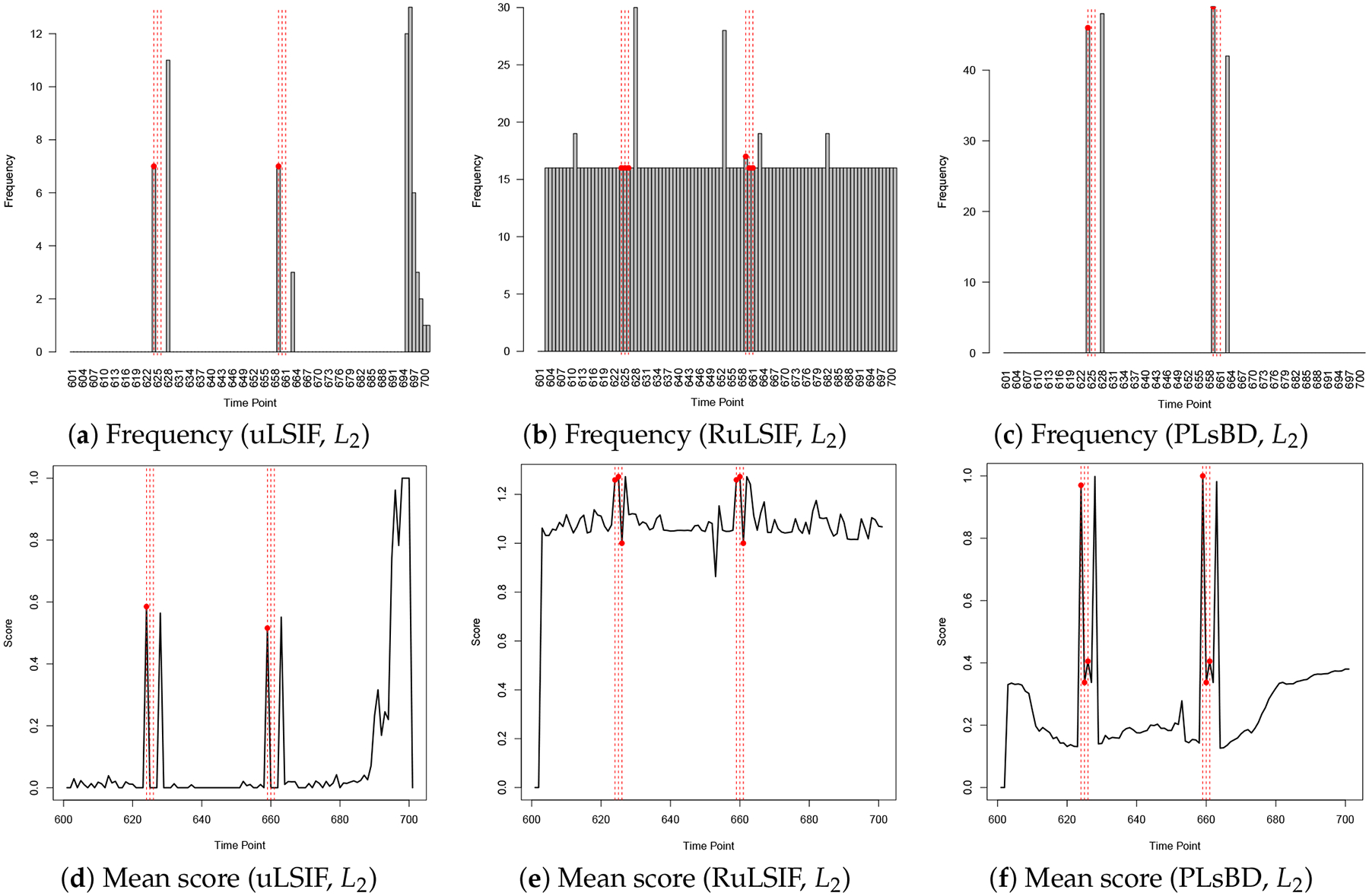
Anomaly detection results in Yahoo Dataset using the uLSIF, RuLSIF, and PLsBD methods with the $L_{2}$ loss function; the mixing parameter α is set to be 0.1 for RuLSIF and PLsBD. The black bars indicate the frequencies that an observation is flagged as an anomaly in 100 runs. Red dashed vertical lines mark the true anomaly indices.

Figure 6 illustrates the frequency of anomaly candidates and their mean divergence scores over 50 runs using uLSIF (a, d), RuLSIF (b, e), and PLsBD (c, f) measures with the $L_{2}$ loss. Since the anomalies are grouped into two clusters (624–626 and 659–661), an anomaly is considered detected if any point within a cluster is identified. The results demonstrate that the PLsBD method, based on scaled Bregman divergence, outperforms other approaches, including uLSIF and RuLSIF, by accurately detecting all anomalies while minimizing false positives. In comparison, the traditional uLSIF method identifies false anomaly intervals, and RuLSIF produces flat mean divergence scores, which fail to reliably detect anomalies and result in numerous false positives. Therefore, our PLsBD method provides a more robust and reliable framework for anomaly detection, particularly in challenging scenarios.

**Figure 7.**
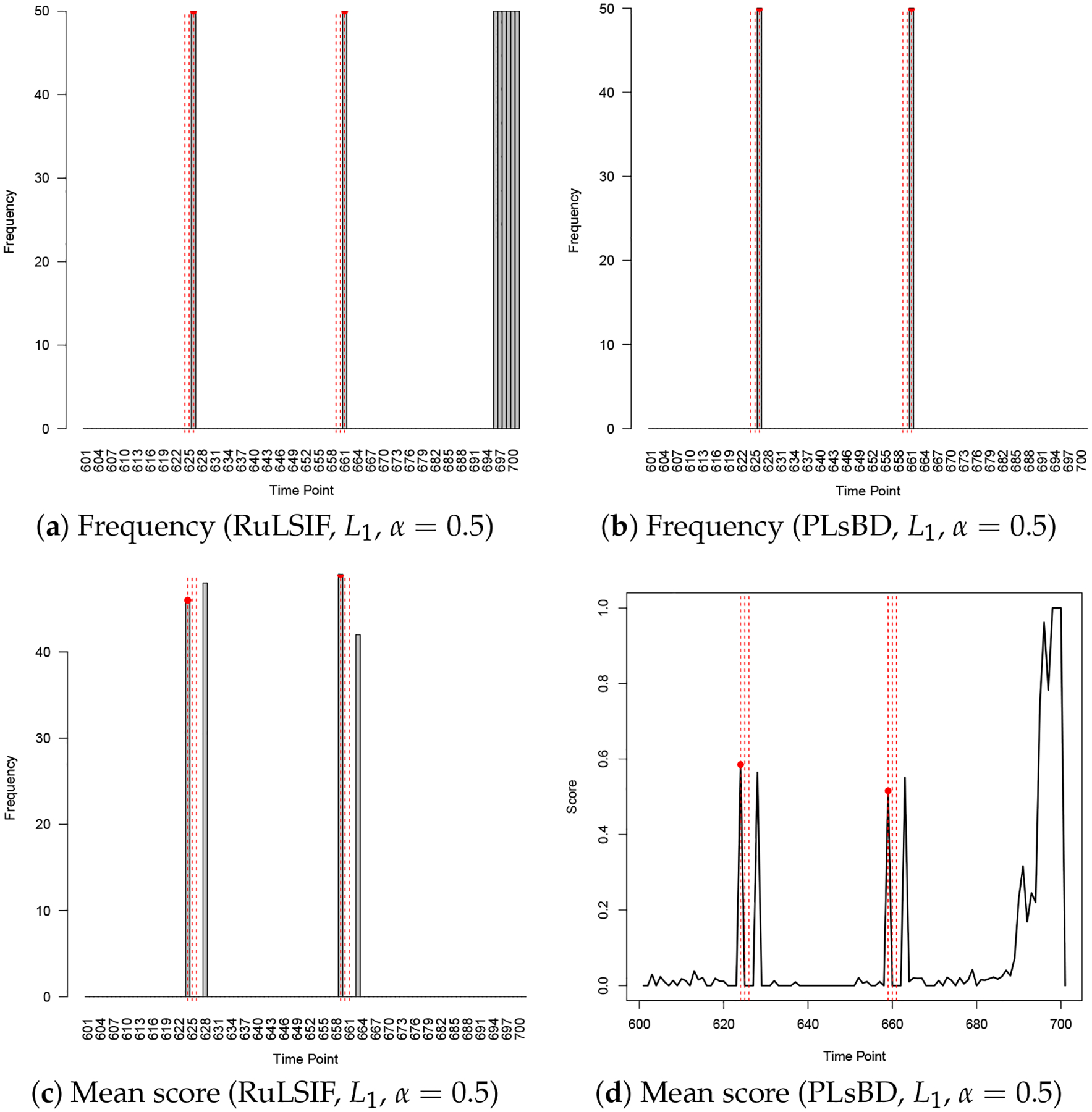
Anomaly detection results in Yahoo Synthetic 11 using the RuLSIF and PLsBD methods with the $L_{1}$ loss function; the mixing parameter α is set to be 0.5. The black bars indicate the frequencies that an observation is flagged as an anomaly in 50 runs. Red dashed vertical lines mark the true anomaly indices.

**Figure 8.**
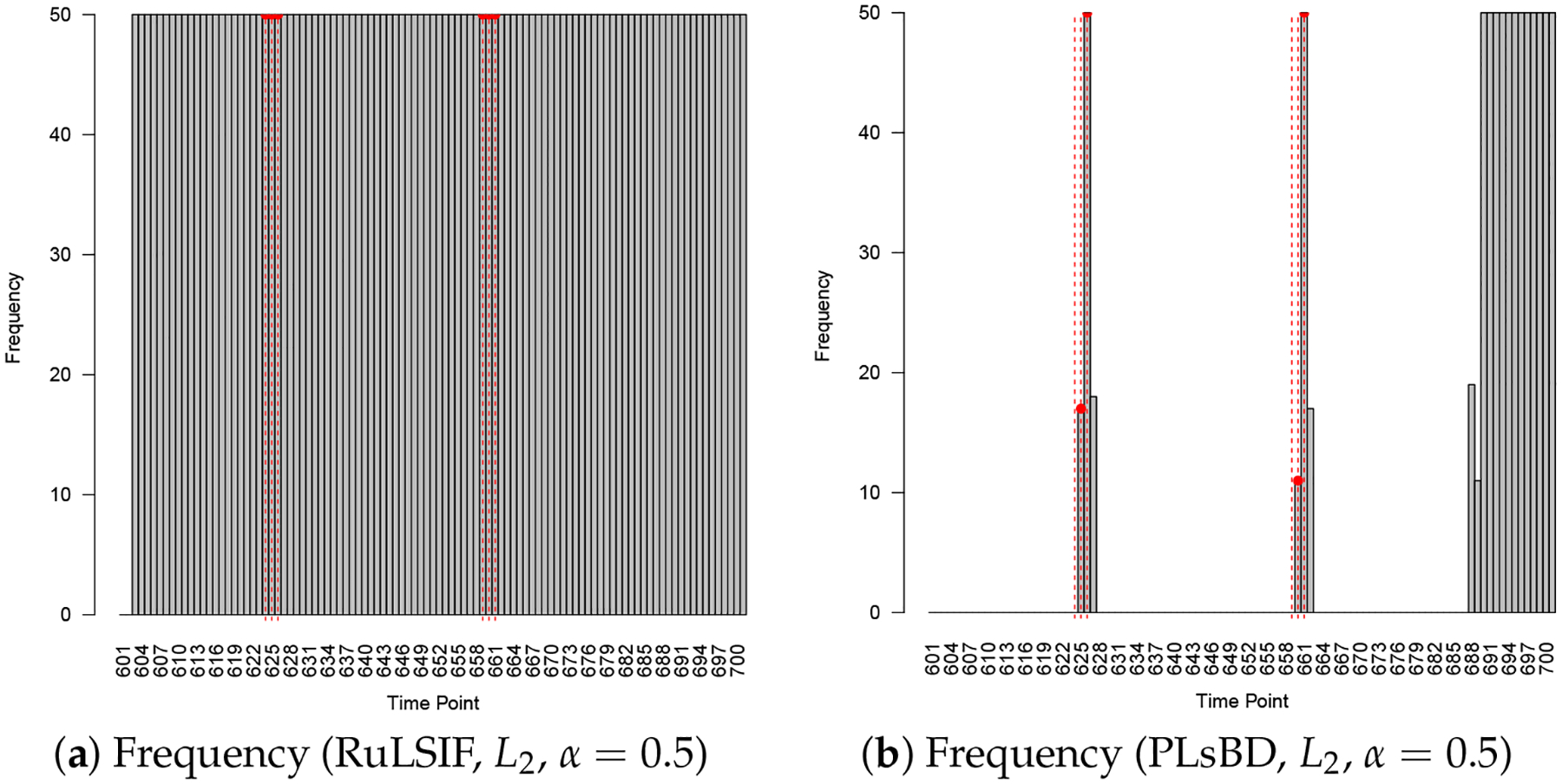

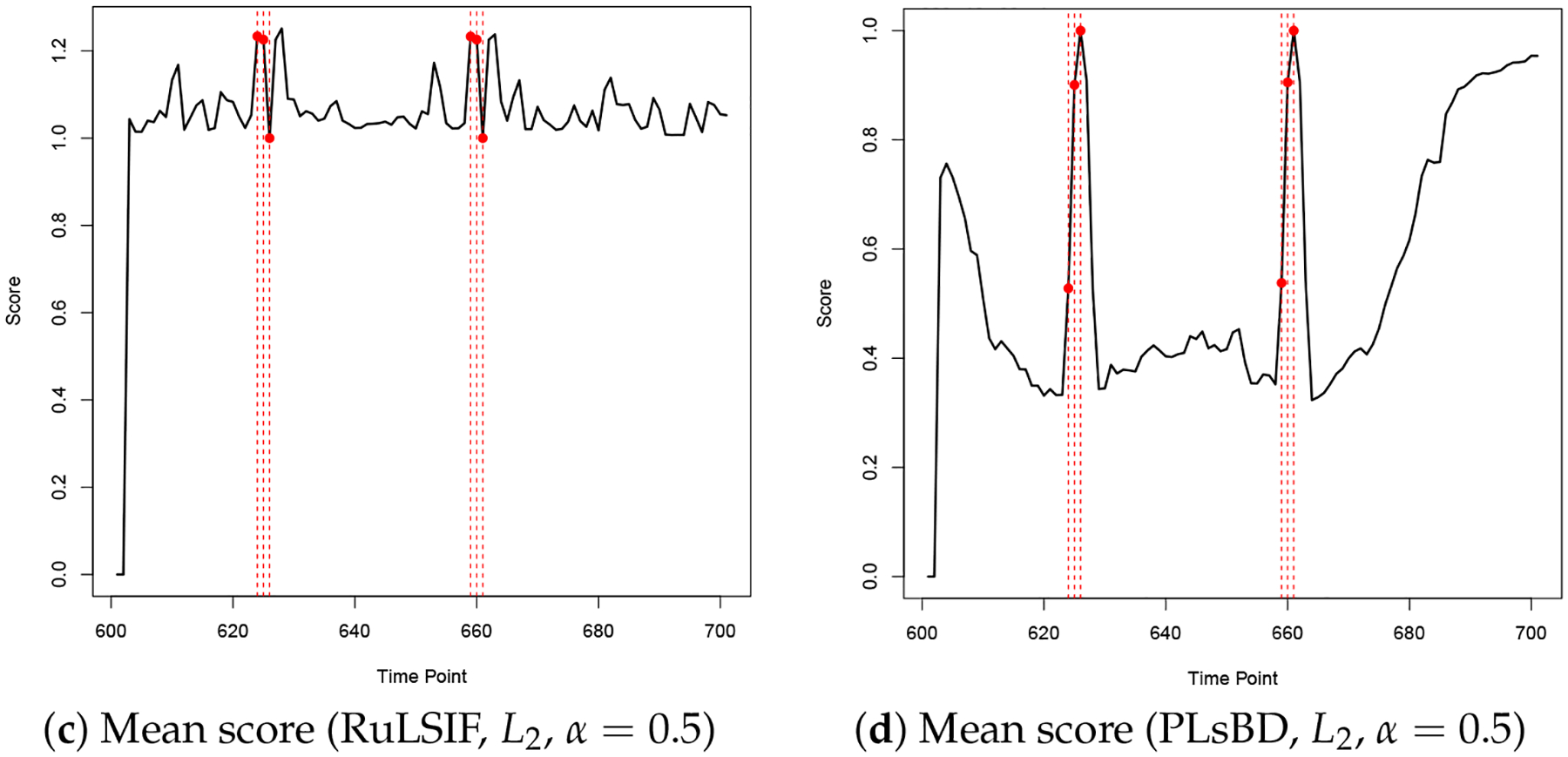
Anomaly detection results in Yahoo Synthetic 11 using the RuLSIF and PLsBD methods with the $L_{2}$ loss function; the mixing parameter α is set to be 0.5. The black bars indicate the frequencies that an observation is flagged as an anomaly in 50 runs. Red dashed vertical lines mark the true anomaly indices.

We increased α from 0.1 to 0.5 and evaluated the performance of RuLSIF and PLsBD using both absolute and squared error loss functions. Figure 7 shows that with the $L_{1}$ loss, both methods detected the two anomaly intervals, but RuLSIF introduced more false positives near the dataset’s end, likely due to increased sensitivity to minor fluctuations. As shown in Figure 8, with squared error loss, PLsBD detected both anomaly intervals but also produced some false positives, while RuLSIF misclassified all points as anomalies, and the mean divergence score was flat, making it ineffective in anomaly detection. These results underscore the importance of selecting appropriate loss functions and the mixing parameter α for effective anomaly detection. Overall, PLsBD outperformed RuLSIF, demonstrating greater robustness and minimal sensitivity to α and the choice of loss functions in this context.

#### High-dimensional Biomedical Benchmark Data.

We consider four biomedical-related datasets, *Diabetes, Heart, BreastCancer, and Thyorid*, available from Rätsch’s benchmark Repository [43]. This benchmark dataset varies in size and complexity and has been widely used to test the performance of different anomaly detection algorithms. Each dataset consists of a feature matrix, where rows represent data points and columns represent features, and a label vector, where (1) indicates normal samples and (−1) denotes anomalies. Table 1 summarizes the number of features and observations for each data point.

In our setup, the training set contains only normal samples (label 1), and the test set includes all normal samples along with a randomly sampled fraction (20%) of anomalies. To ensure a robust evaluation of the anomaly detection algorithms, we performed 20 trials using fixed training and testing splits. For each trial, the window size was set to 10, the step size to 5, and the threshold to 0.9. Other parameters, including the number of kernel functions and the use of 5-fold cross-validation, were kept consistent with the previous data analysis. Algorithm 1 was then applied to identify all potential anomaly candidates using different values of α (0.01, 0.1, 0.5, and 0.9), while also measuring the computation time for each configuration.

To evaluate the performance of anomaly detection algorithms, we use the area under the curve (AUC) of the receiver operating characteristic (ROC) curve, a widely adopted metric in anomaly detection studies. The AUC summarizes a model’s ability to produce relative scores that distinguish between positive and negative instances across all classification thresholds. By capturing the trade-off between the True Positive Rate (TPR) and False Positive Rate (FPR) across thresholds, AUC provides a consistent, threshold-independent measure, making it especially suitable for comparing models in scenarios with imbalanced data.

**Figure 9.**
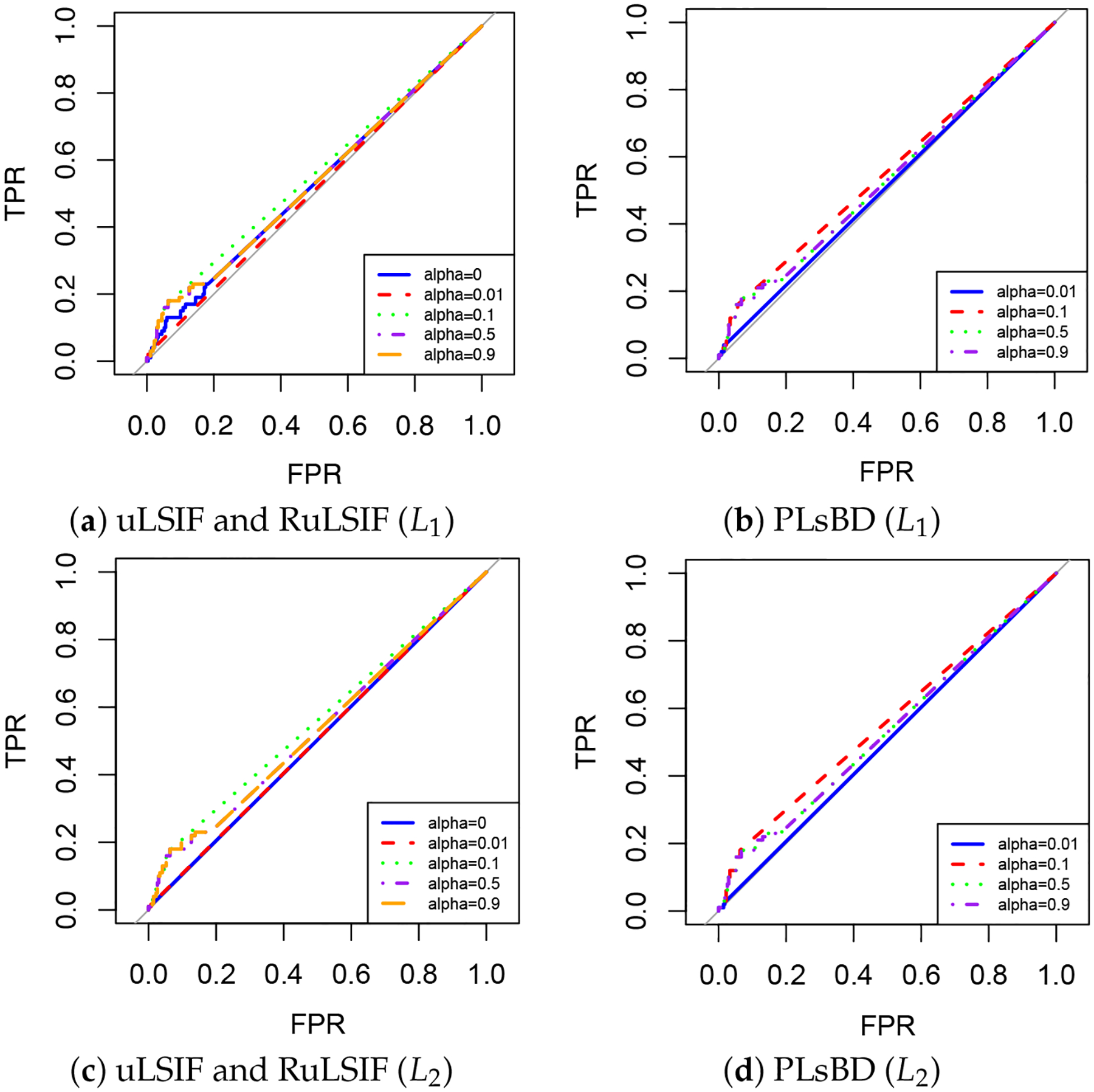
ROC curves for uLSIF, RuLSIF, and PLsBD methods with $L_{1}$ and $L_{2}$ loss functions on the diabetes dataset. Each plot shows results for different mixing parameters α(α=0.01,0.1,0.5,0.9).

Table 2 summarizes the mean AUC values over 20 trials for the diabetes and heart datasets (Table 2), and the ROC curve for the diabetes data is shown in Figure 9. These results were evaluated using different α values and loss functions, along with the corresponding computation times.

The results in Table 2 show that the scaled Bregman divergence-based methods PLsBD and RuLSIF outperform uLSIF across all datasets with the same parameter settings, including both $L_{1}$ and $L_{2}$ loss functions. Previous studies have indicated that the mixing parameter α influences the performance of the algorithm. Table 2 confirms that smaller values of α (e.g., α=0.1) lead to better performance, with larger AUC scores, while larger values of α (e.g., α=0.9) result in decreased performance. For instance, in the diabetes dataset, both RuLSIF and PLsBD achieve the best performance at α=0.1 with AUC values exceeding 0.55 for both $L_{1}$ and $L_{2}$ loss functions. When α=0.5 or higher, the AUC value drops to 0.52. In the simulation data analysis (Figures 1–5), the choice of loss function, whether absolute error loss $\left(L_{1}\right)$ or squared error loss $\left(L_{2}\right)$, significantly impacts the performance of anomaly detection when the number of anomalies is small. However, in the benchmark dataset, where at least 20% of the data points are anomalies, the results in Table 2 show that the difference in performance between the two loss functions is minimal. Table 3 presents the results for the thyroid and breast cancer datasets, showing trends consistent with the findings in Table 2.

We also summarize the computation time for each method with different loss functions and varying values of α in Tables 2 and 3. The results align with the theoretical complexity analysis presented earlier. Specifically, when the loss function is fixed, the computation times across all three anomaly detection methods are comparable. However, methods based on the $L_{2}$ loss function require significantly more computation time than those using $L_{1}$ loss. This is because $L_{2}$ loss involves matrix inversion, which is computationally expensive, whereas $L_{1}$ loss avoids matrix inversion, relying only on element-wise operations and vector additions. Consequently, $L_{1}$ loss offers a more computationally efficient alternative while maintaining comparable performance in this case.

In previous studies, metrics such as accuracy, precision, recall, and F1 score have been widely used to evaluate anomaly detection methods. These metrics are calculated based on true positive (TP), false positive (FP), false negative (FN), and true negative (TN) values. However, their dependency on a chosen threshold can lead to varying conclusions, making them less reliable for consistent model comparison. To provide a more comprehensive evaluation, we also computed accuracy, precision, F1 score, and computation time for different loss functions and varying values of α. The results, summarized in Table 4, further validate that RuLSIF and PLsBD methods favor smaller α values, with computation time primarily influenced by the chosen loss function.

## 4. Discussion

In this work, we proposed and applied a novel scaled Bregman divergence-based anomaly detection algorithm to identify abnormal observations in both low- and high-dimensional data. Compared to existing density ratio estimation-based anomaly detection algorithms, such as KLIEP and uLSIF, our method addresses the unboundedness issue. Additionally, we derived an analytical formula to learn the density ratio using both the least absolute error and least squared error as loss functions. We applied the scaled Bregman divergence-based method, PLsBD, along with RuLSIF and uLSIF, to identify anomalies in both simulation data and real-world high-dimensional data, comparing their performance. Our results demonstrate that both PLsBD and RuLSIF outperform uLSIF, which was previously considered the state-of-the-art density ratio-based anomaly detection method. We also found that the mixing parameter α, window size, and step size significantly influence the algorithm’s performance, with smaller α values (e.g., α=0.1) and smaller step sizes yielding better results. Moreover, when the data contain fewer anomalies, the $L_{2}$ loss function outperforms the $L_{1}$ loss. However, as the number of anomalies increases, the performance difference between the two loss functions becomes minimal. Both theoretical complexity analysis and real-world data evaluations demonstrate that the $L_{1}$ loss-based method is more computationally efficient than the $L_{2}$ loss-based methods, while maintaining comparable performance in high-dimensional data analysis.

Although the proposed scaled Bregman divergence-based anomaly detection algorithm effectively resolves the unboundedness issue and outperforms the uLSIF method; however, it has several limitations. Currently, the method assumes that the input data are complete and does not account for missing values. However, many datasets may contain missing values, necessitating the use of imputation or pre-processing techniques to handle incomplete observations. If not applied carefully, these methods can introduce biases or inaccuracies. Moreover, the proposed method involves several pre-processing steps, such as kernel matrix computation and parameter optimization, which can be computationally intensive for high-dimensional data or large datasets. These steps may also require careful tuning to ensure robustness and effectiveness. To address these limitations, future work could incorporate advanced imputation methods [44–47] and develop more efficient computational techniques to streamline the pre-processing pipeline. This integration with the scaled Bregman divergence framework could enhance robustness and scalability, enabling effective anomaly detection in high-dimensional datasets with missing values.

Recently, deep learning-based methods, including GAN-based [19,20,23,48], VAE-based [21,22,49], transformer-based [24,25,50], and diffusion model-based [27,51,52] anomaly detection techniques, have gained significant popularity. These approaches excel in capturing complex patterns within high-dimensional datasets, modeling intricate data distributions, and effectively identifying subtle anomalies. However, these methods typically require a large amount of labeled data for effective training, which can be a significant limitation in scenarios where labeled data are scarce. Additionally, deep learning approaches are computationally intensive due to the need for training Deep Neural Networks, which involves iterative gradient-based optimization over a large number of parameters. This computational demand makes them less practical for real-time or resource-constrained applications. In contrast, our proposed method is computationally more efficient, requiring only a small number of parameters to tune. So, although deep learning methods may be better suited for scenarios with abundant computational resources and well-annotated datasets, our method offers a competitive alternative in settings with limited computational capacity or scarce labeled data.

## Figures and Tables

**Table 1.** Summary of biomedical datasets.

Dataset	Diabetes	Heart	Thyroid	Breast Cancer
**Number of Features**	8	13	5	9
**Number of Observations**	768	270	215	263

**Table 2.** Mean AUC values for the diabetes and heart datasets, evaluated with different α values and loss functions, including computation time.

Method	Loss	α	Diabetes	Time (s)	Heart	Time (s)
uLSIF	L1	0	0.5246	74.28	0.4847	27.97
	L2	0	0.5031	92.60	0.4917	32.14
RuLSIF	L1	0.01	0.5088	74.26	0.4925	24.58
	L1	0.10	0.5546	75.04	0.5310	24.35
	L1	0.50	0.5236	73.91	0.4928	24.52
	L1	0.90	0.5236	74.52	0.4928	24.68
	L2	0.01	0.5043	93.31	0.4920	31.99
	L2	0.10	0.5565	92.95	0.5193	31.82
	L2	0.50	0.5236	94.05	0.4931	32.22
	L2	0.90	0.5236	92.80	0.4919	32.37
PLsBD	L1	0.01	0.5107	71.33	0.5036	25.08
	L1	0.10	0.5520	71.25	0.5497	23.19
	L1	0.50	0.5236	70.94	0.4936	23.56
	L1	0.90	0.5236	70.13	0.4933	22.52
	L2	0.01	0.5037	90.47	0.4917	30.68
	L2	0.10	0.5580	89.70	0.5210	30.50
	L2	0.50	0.5236	89.81	0.4925	30.80
	L2	0.90	0.5236	89.55	0.4931	30.67

**Table 3.** Mean AUC values of 20 for thyroid and breast cancer datasets with different α values and loss functions, including computation time.

Method	Loss	α	Thyroid	Time (s)	Breast Cancer	Time (s)
uLSIF	L1	0	0.4949	14.57	0.4180	19.99
	L2	0	0.4769	18.71	0.5065	37.65
RuLSIF	L1	0.01	0.4985	13.50	0.4260	18.99
	L1	0.10	0.5318	13.53	0.5002	17.89
	L1	0.50	0.5231	13.59	0.4446	18.66
	L1	0.90	0.5108	14.75	0.4207	18.86
	L2	0.01	0.4769	18.24	0.5065	27.42
	L2	0.10	0.5495	18.30	0.5002	23.87
	L2	0.50	0.5241	18.37	0.4619	25.69
	L2	0.90	0.5123	18.56	0.4270	23.37
PLsBD	L1	0.01	0.4615	12.70	0.4812	16.25
	L1	0.10	0.4846	13.13	0.5002	16.93
	L1	0.50	0.5277	13.14	0.4506	16.03
	L1	0.90	0.5103	13.15	0.4204	16.07
	L2	0.01	0.4769	17.47	0.5065	21.64
	L2	0.10	0.5338	17.88	0.5002	22.79
	L2	0.50	0.5223	17.77	0.4439	21.87
	L2	0.90	0.5108	17.40	0.4207	24.81

**Table 4.** Performance metrics (accuracy, precision, F1 score) and computation time for the diabetes dataset analysis.

Method	Loss	α	Accuracy	Precision	F1 Score	Time (s)
uLSIF	L1	0.00	0.6591	0.3219	0.2683	73.56
	L2	0.00	0.7283	0.5000	0.0196	91.56
RuLSIF	L1	0.01	0.7228	0.4000	0.0727	73.73
	L1	0.10	0.7255	0.4865	0.2628	73.59
	L1	0.50	0.6576	0.3194	0.2674	72.54
	L1	0.90	0.6576	0.3194	0.2674	72.18
	L2	0.01	0.7204	0.3400	0.0568	95.74
	L2	0.10	0.7284	0.5007	0.2648	94.14
	L2	0.50	0.6576	0.3194	0.2674	94.61
	L2	0.90	0.6576	0.3194	0.2674	94.28
PLsBD	L1	0.01	0.7255	0.4444	0.0734	71.79
	L1	0.10	0.7310	0.5161	0.2443	70.94
	L1	0.50	0.6576	0.3194	0.2674	69.72
	L1	0.90	0.6576	0.3194	0.2674	70.40
	L2	0.01	0.7213	0.3583	0.0497	89.53
	L2	0.10	0.7308	0.5136	0.2660	90.84
	L2	0.50	0.6576	0.3194	0.2674	91.69
	L2	0.90	0.6576	0.3194	0.2674	89.15

## Data Availability

The original Yahoo data presented in the study are available from Yahoo at https://webscope.sandbox.yahoo.com/ (accessed on 15 January 2025). The original Biomedical Benchmark data are openly available in Zenodo at https://zenodo.org/records/18110 (accessed on 15 January 2025). The R code for the proposed method is available on GitHub: https://github.com/yungewang/AnomalyDetection-by-Density-Ratio-Estimation (accessed on 15 January 2025).

## Abbreviations

The following abbreviations are used in this manuscript:

PLsBD: Pearson-like scaled Bregman divergence
RuLSIF: Relative unconstrained least-squares importance fitting
uLSIF: Unconstrained least-squares importance fitting
ROC: Receiver operating characteristic
AUC: Area under the curve
